# Unsupervised layer-wise feature extraction algorithm for surface electromyography based on information theory

**DOI:** 10.3389/fnins.2022.975131

**Published:** 2022-08-16

**Authors:** Mingqiang Li, Ziwen Liu, Siqi Tang, Jianjun Ge, Feng Zhang

**Affiliations:** ^1^Information Science Academy, China Electronics Technology Group Corporation, Beijing, China; ^2^School of Mathematical Sciences, University of Chinese Academy of Sciences, Beijing, China

**Keywords:** information theory, feature extraction, unsupervised learning, information bottleneck, disentangled representation, surface electromyography

## Abstract

Feature extraction is a key task in the processing of surface electromyography (SEMG) signals. Currently, most of the approaches tend to extract features with deep learning methods, and show great performance. And with the development of deep learning, in which supervised learning is limited by the excessive expense incurred due to the reliance on labels. Therefore, unsupervised methods are gaining more and more attention. In this study, to better understand the different attribute information in the signal data, we propose an information-based method to learn disentangled feature representation of SEMG signals in an unsupervised manner, named Layer-wise Feature Extraction Algorithm (LFEA). Furthermore, due to the difference in the level of attribute abstraction, we specifically designed the layer-wise network structure. In TC score and MIG metric, our method shows the best performance in disentanglement, which is 6.2 lower and 0.11 higher than the second place, respectively. And LFEA also get at least 5.8% accuracy lead than other models in classifying motions. All experiments demonstrate the effectiveness of LEFA.

## Introduction

Feature engineering is an important component of pattern recognition and signal processing. Learning good representations from observed data can help reveal the underlying structures. In recent decades, feature extraction methods (He et al., 2016; Howard et al., 2017; Hassani and Khasahmadi, 2020; Zbontar et al., 2021) have drawn considerable attention. Due to the high cost of obtaining labels, supervised learning methods suffer from data volume limitations. Unsupervised learning methods therefore becomes critical for feature extraction. Most of these are based on probabilistic models, such as maximum likelihood estimation (Myung, 2003), maximum *a posteriori* probability estimation (Richard and Lippmann, 1991), and mutual information (MI) (Thomas and Joy, 2006). Methods such as principal component analysis (PCA) (Abdi and Williams, 2010), linear discriminant analysis (Izenman, 2013), isometric feature mapping (Tenenbaum et al., 2000), and Laplacian eigenmaps (Belkin and Niyogi, 2003) are widely used owing to their good performance, high efficiency, flexibility, and simplicity. Other algorithms are based on reconstruction errors or generative criteria, such as autoencoders (Bengio et al., 2013) and generative adversarial networks (GANs) (Goodfellow et al., 2014). Occasionally, the reconstruction error criterion also has a probabilistic interpretation.

In recent years, deep learning has become a dominant method of representation learning, particularly in the supervised case. A neural network simulates the mechanism of hierarchical information processing in the brain and is optimized using the back propagation (BP) algorithm (LeCun et al., 1988). Because several feature engineering tasks are unsupervised, that is, no label information is available in the real situation and collecting considerable labeled data is expensive, methods to discover the feature representation in an unsupervised case have been significantly developed in recent years. MI maximization (Bell and Sejnowski, 1995) and minimization criteria (Matsuda and Yamaguchi, 2003) are powerful tools for capturing salient features of data and disentangling these features. In particular, variational autoencoder (VAE) (Kingma and Welling, 2013) based models and GAN have exhibited effective applications in disentangled representations. There are two benefits of learning disentangled representations. First, models with disentangled representations are more explainable (Bengio et al., 2013; Liu et al., 2021). Second, disentangled representations make it easier and more efficient to manipulate training-data synthesis. However, the backpropagation algorithm still requires a high amount of computation and data.

To extract features information in SEMG signal data, we propose a Layer-wise Feature Extraction Algorithm (LFEA) based on information theory in the unsupervised case, which includes a hierarchical structure to capture disentangled features. In each layer, we split the feature into two independent blocks, and ensure the information separation between the blocks *via* information constraint, which we called Information Separation Module (ISM). Moreover, to ensure the expressiveness of the representation without losing crucial information, we propose the Information Representation Module (IRM) to enable the learned representation to reconstruct the original signal data. Meanwhile, redundant information would affect the quality of the representation and thus degrade the effectiveness of downstream tasks, for which Information Compression Module (ICM) is proposed to reduce the redundant and noisy information. In terms of the optimization algorithm, our back-propagation process is only performed in a single layer and not back propagated throughout the network, which can greatly reduce the amount of computation while having no effect on the effectiveness of our method. Regarding the experiments, we have made improvement and strengths in terms of motion classification and representation disentanglement over the traditional methods of surface electromyography (SEMG). Especially, on NinaPro database 2 (DB2) dataset, our approach gets a significant 4% improvement in the motion classification, and better model stability.

This manuscript is organized as follows. In Section 2, we introduce the related work. The proposed method LFEA is described in Section 3. We present the numerical results in Section 4. Section 5 gives the conclusion of this manuscript.

## Related work

### Disentangled representation

The disentanglement problem has played a significant role, particularly because of its better interpretability and controllability. The VAE variants construct representations in which each dimension is independent and corresponds to a dedicated attribute. β-VAE (Higgins et al., 2016) adds a hyperparameter to control the trade-off between compression and expression. An analysis of β-VAE by Burgess et al. (2018) is provided, and the capacity term is proposed to obtain a better balance of the reconstruction error. Penalizing the total correlation term to reinforce the independence among representation dimensions was proposed in Factor VAE (Kim and Mnih, 2018) and β-TCVAE (Chen et al., 2018). FHVAE (Hsu et al., 2017) and DSVAE (Yingzhen and Mandt, 2018) constructed a new model architecture and factorized the latent variables into static and dynamic parts. Cheng et al. (2020b) described a GAN model using MI. Similar to our study, Gonzalez-Garcia et al. (2018) proposed a model to disentangle the attributes of paired data into shared and exclusive representations.

### Information theory

Shannon’s MI theory (Shannon, 2001) is a powerful tool for characterizing good representation. However, one major problem encountered in the practical application of information theory is computational difficulties in high-dimensional spaces. Numerous feasible computation methods have been proposed, such as Monte Carlo sampling, population coding, and the mutual information neural estimator (Belghazi et al., 2018). In addition, the information bottleneck (IB) principle (Tishby et al., 2000; Tishby and Zaslavsky, 2015; Shwartz-Ziv and Tishby, 2017; Jeon et al., 2021) learns an informative latent representation of target attributes. A variational model to make IB computation easier was introduced in variational IB (Alemi et al., 2016). A stair disentanglement net was proposed to capture attributes in respective aligned hidden spaces and extend the IB principle to learn a compact representation.

### Surface electromyography signal feature extraction

With the development of SEMG signal acquisition technology, the analysis and identification of SEMG signals has also drawn the attention of researchers.

As machine learning has demonstrated excellent feature extraction capabilities in areas such as images and speech, it can also be a good solution for recognizing SEMG signals. The basic motivation was to construct and simulate neural networks for human brain analysis and learning. Deep neural networks can extract the features of SEMG signals while effectively avoiding the absence of valid information in the signal and improving the accuracy of recognition. Xing et al. (2018) used a parallel architecture model with five convolutional neural networks to extract and classify SEMG signals. Atzori et al. (2016) used a convolutional network to classify an average of 50 hand movements from 67 intact subjects and 11 transradial amputees, achieving a better recognition accuracy than traditional machine learning methods. Zhai et al. (2017) proposed a self-calibrating classifier. This can automatically calibrate the original classifier. The calibrated classifier also obtains a higher accuracy than the uncalibrated classifier. In addition, He et al. (2018) incorporated a long short-term memory network (Hochreiter and Schmidhuber, 1997) into a multilayer perceptron and achieved better classification of SEMG signals in the NinaPro DB1 dataset.

As stated, deep learning methods can help overcome the limitations of traditional methods and lead to better performance of SEMG. Furthermore, deep-learning methods can provide an extensive choice of models to satisfy different conditional requirements.

## Method

### Preliminary

Information theory is commonly used to describe stochastic systems. Among the dependency measurements, mutual information (MI) was used to measure the correlation between random variables or factors. Given two random variables *X* and *Z*, the MI is defined as follows:


(1) $$I\,\left(X;Z\right)=E_{p\,\left(x,z\right)}\,\left[\log\frac{p\,\left(x,z\right)}{p\,\left(x\right)\,p\,\left(z\right)}\right]$$

Regarding the data processing flow as a Markov chain *X*→*Z*→*Y*, the information bottleneck (IB) principle desires that the useful information in the input X can pass through the ‘bottleneck’ while the noise and irrelevant information are filtered out. The IB principle is expressed as follow:


(2) $$\min\,R_{\mathrm{IB}}=I\,\left(X;Z\right)-\beta \,I\,\left(Z;Y\right)$$

where, β is the tradeoff parameter between the complexity of the representation and the amount of relevant essential information.

### Framework

**FIGURE 1:**
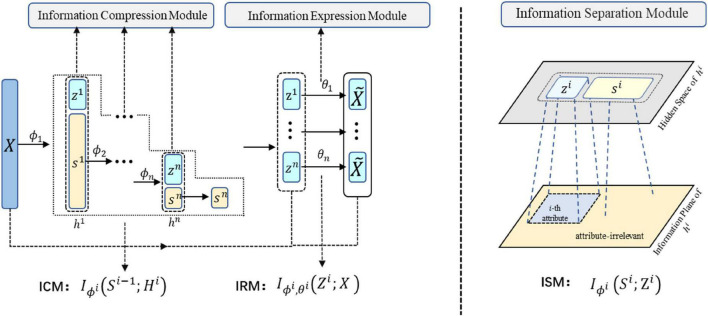
The diagram of Layer-wise Feature Extraction Algorithm (LFEA). LEFA contains three core modules: Information Compression Module (ICM), Information Expression Module (IEM) and Information Separation Module (ISM), to ensure compression, expression and disentanglement of representation, respectively.

The diagram of our proposed Layer-wise Feature Extraction Algorithm (LFEA) is illustrated in Figure 1. Our algorithm aims to learn a representation that satisfies three main properties: “Compression,” “Expression” and “Disentanglement.” To this end, three key information process modules are introduced, including the information compression module (ICM), information expression module (IEM), and information separation module (ISM) in each layer.

In the ICM, input *s*^*i*−1^ of layer *i* is compressed into *h*^*i*^ (*s*^0^ = *X*). In the IEM, *z*^*i*^ as part of *h*^*i*^ is constrained to represent the original input *X*. In the ISM section, *s*^*i*^ and *z*^*i*^ are irrelevant. The parameters of the ICM and IEM in layer *i* are denoted as ϕ*^i^* and θ*^i^*. The data information flow can be expressed as follows:


(3) $$h^{i}∼q_{\phi^{i}}\,\left(h^{i}|s^{i-1}\right),$$

(4) $$h^{i}=\left(z^{i},s^{i}\right),$$

(5) $$\tilde{X}∼p_{\theta^{i}}\,\left(\tilde{X}|z^{i}\right),$$

where, *s*^0^ = *X*, and $q_{\phi^{i}}$ and $p_{\theta^{i}}$ are the condition distributions with ϕ*^i^* and θ*^i^* for *h*^*i*^ and $\tilde{X}$. In following sections, we describe these three modules in detail.

### Information compression module

According to (3), *h*^*i*^ is the hidden representation of *s*^*i*−1^. To ensure information ‘compression,’ the optimal representation of *s*^*i*−1^ should forget redundant information altogether, that is, *h*^*i*^ represents *s*^*i*−1^ with the lowest bits. Formally, the objective in the *i*-th layer to be minimized is as follows:


(6) $$m\,i\,n\,L_{I\,C\,M}≜I_{\phi^{i}}\,\left(S^{i-1};H^{i}\right)$$

Due to intractability of mutual information, optimizing *L*_*ICM*_ with gradient methods directly is not feasible. We therefore derived the upper bound of *L*_*ICM*_ with the variational inference method and get decomposition as follows:


(7) $$I_{\phi^{i}}\,\left(S^{i-1};H^{i}\right)=E_{q_{\phi^{i}}\,\left(s^{i-1},h^{i}\right)}\,\left[\log\frac{q_{\phi^{i}}\,\left(h^{i}|s^{i-1}\right)\,p\,\left(h\right)}{q_{\phi^{i}\,\left(h^{i}\right)}\,p\,\left(h\right)}\right]=L_{\mathrm{ICM}}^{\mathrm{upper}}-D_{\mathrm{KL}}\left(q_{\phi^{i}}\left(h^{i}\right)||p\left(h\right)\right),$$

where, *p*(*h*) is the prior, and $L_{\mathrm{ICM}}^{\mathrm{upper}}$ is the upper bound of *L*_*ICM*_ defined as follows:


(8) $$L_{\mathrm{ICM}}^{\mathrm{upper}}=E_{q_{\phi^{i}}\,\left(s^{i-1}\right)}\left[D_{\mathrm{KL}}\left(q_{\phi^{i}}\left(h^{i}|s^{i-1}\right)||p\left(h\right)\right)\right],D_{\mathrm{KL}}\,\left(P,Q\right)={\text{E}}_{\text{P}}\,\left[\log\frac{\text{p}}{\text{q}}\right].$$

### Information expression module

With the ICM guaranteeing the information compression, LFEA also need to ensure the expressiveness of the representation to the data. We therefore propose the information expression module (IEM). To ensure sufficient information to reconstruct the original data *X*, we maximize the MI between and *Z*^*i*^ in *i*-th layer, that is,


(9) $$\text{m}\,a\,x\,{\text{L}}_{I\,E\,M}≜{\text{I}}_{\phi^{i},\theta^{i}}\,\left({\text{z}}^{i};X\right)$$

For *L*_*IEM*_, we can obtain a lower bound using the variational approximation method as follows:


(10) $$L_{\mathrm{IEM}}\geq L_{\mathrm{IEM}}^{\mathrm{lower}}-D_{\mathrm{KL}}\left(p\left(x\right)||p_{\theta^{i}}\left(x\right)\right),$$

where, $p_{\theta^{i}}\,\left(x\right)$


(11) $$L_{\mathrm{IEM}}^{\mathrm{lower}}=E_{p\,\left(\text{x}\right)}\,\left[E_{q_{\phi^{i}}\,\left(z^{i}|x\right)}\,\log p_{\theta^{i}}\,\left(x|z^{i}\right)\right]$$

can be viewed as the reconstruction loss.

### Information separation module

To achieve disentanglement of representations (Independent of each block *z*^1^,*z*^2^,…, *z*^*n*^ in *Z*), we further introduce the information separation module (ISM) in each layer. In *i*-th layer, the principle of ISM is to ensure that there is no intersection information between *z*^*i*^ and *s*^*i*^, that is,


(12) $$\text{m}\,a\,x\,{\text{L}}_{I\,S\,M}≜{\text{I}}_{\phi^{i}}\,\left({\text{z}}^{i};{\text{s}}^{i}\right)=D_{\mathrm{KL}}\left(q_{\phi^{i}}\left(h^{i}\right)||q_{\phi^{i}}\left(z^{i}\right)q_{\phi^{i}}\left(s^{i}\right)\right).$$

**FIGURE 2:**
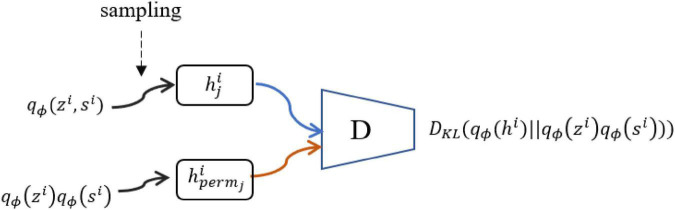
Discriminator *D*(.). To compute and optimize *L*_*ISM*_, we need an additional discriminator as shown in Eq. (13).

In practice, the products of $q_{\phi^{i}}\,\left(z^{i}\right)$ and $q_{\phi^{i}}\,\left(s^{i}\right)$ are not analytical in nature. We introduce discriminator $\hat{D}\left(.\right)$ (see Figure 2) to distinguish samples from the joint distribution and the product of the marginal distribution, that is,


(13) $$L_{\mathrm{ISM}}\approx L_{\mathrm{IEM}}^{e}=E_{q_{\phi^{i}}\,\left(h^{i}\right)}\,\left[l\,o\,g\,\frac{D\left(.\right)}{1-D\left(.\right)}\right].$$

### Algorithm optimization

As presented above, our model contains three modules: ICM, IEM, and ISM. However, during optimization, the back-propagation algorithm is computationally intensive and potentially problematic when training deep networks, so we propose a layer-wise training step. After training one layer of the network, we fix the parameters of the trained layers and only train the next layer in the next step. Finally, we can obtain the final model after training all the layers. Such optimization design allows for training parameters at the bottom layers without bac-propagation from the top layers, avoiding the problems that often occur with deep network optimization, like vanishing and exploding gradient.

## Numerical results

### Dataset

**FIGURE 3:**
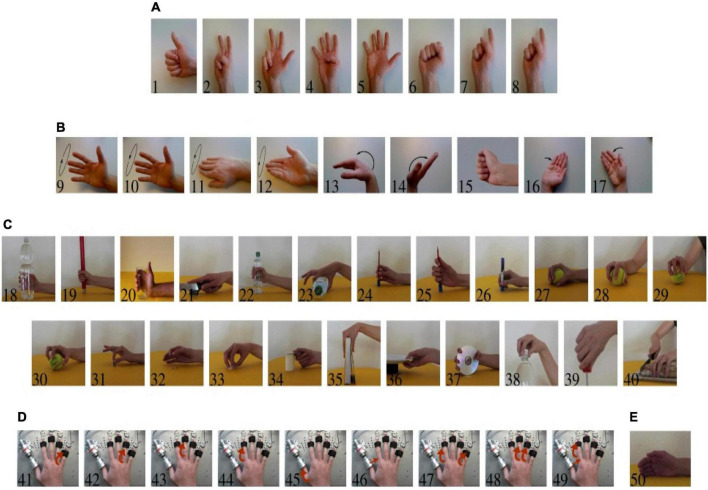
Movements in NinaPro DB2. **(A)** Isometric, isotomic hand configurations. **(B)** Basic movements of the wrist. **(C)** Grasps and functional movements. **(D)** Single and multiple fingers force measurement patterns. **(E)** Rest position. Available from: http://ninapro.hevs.ch/node/123.

In our experiments, we used the NinaPro* DB2 dataset and DB5 dataset. Atzori et al. (2014); Gijsberts et al. (2014) as the benchmark to perform numerical comparisons. NinaPro is a standard dataset for the gesture recognition of sparse multichannel SEMG signals. The SEMG signals in DB2 were obtained from 40 subjects and included 49 types of hand movements (see Figure 3).

**FIGURE 4:**
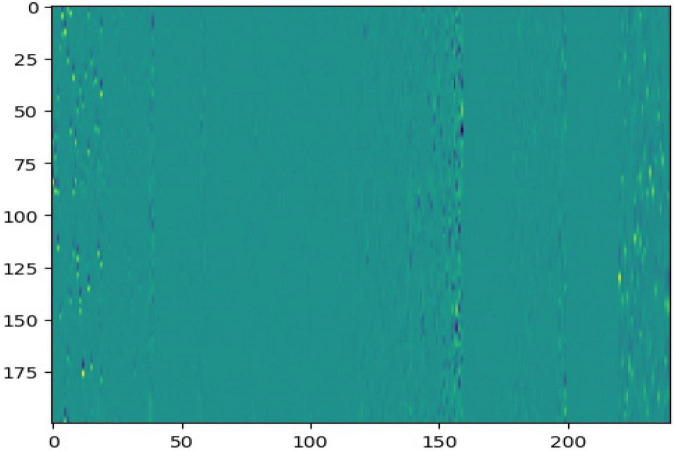
Sample data image.

Detailed attribute information of the five subjects in NinaPro DB2 is shown in Table 1. The original SEMG signal was processed through sliding windows, and the size of the sample data used in the experiment was (200,12). Figure 4 shows 20 processed data points.

**TABLE 1:** Subject attribute information of NinaPro DB2 dataset.

Subject	Hand	Laterality	Gender	Age	Height (cm)	Weight (kg)
1	Intact	Right Handed	Male	29	187	75
2	Intact	Right Handed	Male	29	183	75
3	Intact	Right Handed	Male	31	174	69
4	Intact	Left Handed	Female	30	154	50
5	Intact	Right Handed	Male	25	175	70

**FIGURE 5:**
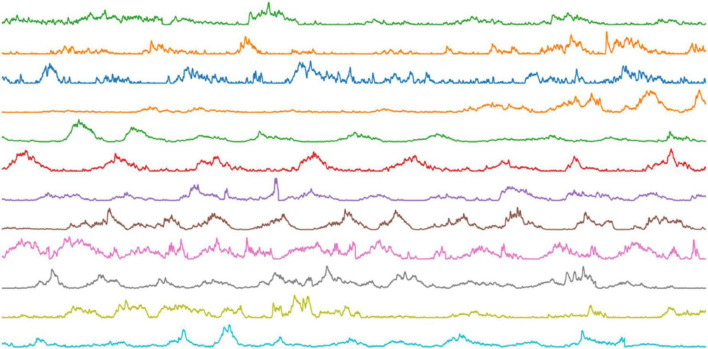
12 basic movements signal of fingers in Exercise A.

DB1 consists of 11 subjects and the data set of each subject contains three types of gestures, which are Exercise A, Exercise B, and Exercise C. Exercise A includes 12 basic movements of fingers (see Figure 5). Exercise B includes 17 movements. Exercise C includes 23 grasping and functional movements.

We preprocessed the dataset with the digital filter to cutoff frequency and sliding window to split signal, which follows He et al. (2018).

### Model setting

In the following experiments, we used four layers model. The loss function is as follows:

$$\min L≜{\text{L}}_{I\,C\,M}^{u\,p\,p\,e\,r}-\lambda \,{\text{L}}_{I\,E\,M}^{l\,o\,w\,e\,r}+\beta \,{\text{L}}_{I\,S\,M},$$

Detail parameters are listed in Table 2.

**TABLE 2:** Detail parameters for LFEA.

Parameter	Value
Number of layers	4
Size of *z*^*i*^	5
λ	0.1
β	0.2

### Results

First, we used total correlation (TC) as the quantitative metric for the quality of the disentanglement of the representation. TC is defined as follows:

$$\mathrm{TC}\,\left(z^{1},z^{2},z^{3},z^{4}\right)=E_{p\,\left(z^{1},z^{2},z^{3},z^{4}\right)}\,\left[\log\,\frac{p\,\left(z^{1},z^{2},z^{3},z^{4}\right)}{p\,\left(z^{1}\right)\,p\,\left(z^{2}\right)\,p\,\left(z^{3}\right)\,p\,\left(z^{4}\right)}\right].$$

The TC was estimated using a three-like algorithm (Cheng et al., 2020a). A low TC score indicated that the representation had less variance. MIG metric (Chen et al., 2018) is another disentanglement metric; the higher the value, the more disentangled representation is. We compared the quality of disentanglement among PCA, β-VAE, VAE, and HFEA. Table 3 shows the comparison results on TC score and MIG metric. In TC score and MIG metric, HFEA has the best performance, which is 6.2 lower and 0.11 higher than the second place, respectively.

**TABLE 3:** Results of TC score.

Method	TC score	MIG
LFEA (Ours)	**12.3**	**0.72**
VAE	23.6	0.54
β-VAE	25.8	0.61
PCA	18.5	0.49

We compare our method the classic methods including VAE, β-VAE and PCA. Our HFEA method is much better than others. The bold indicates the best results.

**FIGURE 6:**
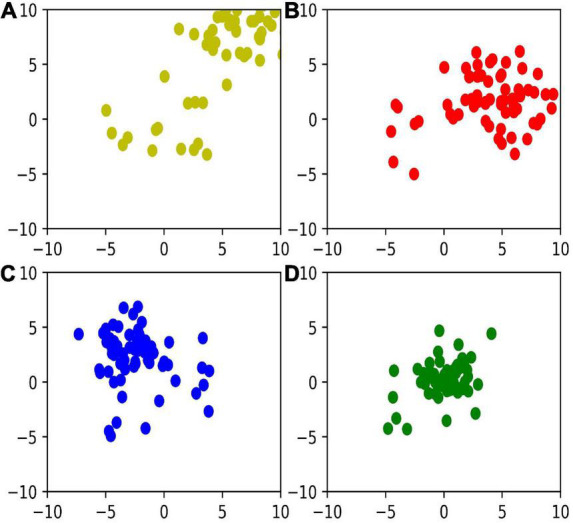
Feature distribution in layer 1–4 with **(A–D)**.

Furthermore, in Figure 6, we visualize the distribution of *z*^1^,*z*^2^,*z*^3^, and *z*^4^, respectively in a two-dimensional space based on t-distributed stochastic neighbor embedding. We can find that the variance of representation decreases with deeper layers, which indicates that the deeper networks learn more robust representations.

Classification results on NinaPro DB2 dataset is described in Table 4. Our method is based on LFEA and SVM and the feature *Z* used in SVM is computed by LFEA.

$$Z=\left(z^{1},z^{2},z^{3},z^{4}\right)$$

**TABLE 4:** Classification results on NinaPro DB2 dataset.

Methods	Windowing	Train/Test	Accuracy
LFEA + SVM(Ours)	200 ms	2/1	75.2 ± 2.3%
CNN	200 ms	2/1	65.7 ± 5.9%
LSTM + MLP	200 ms	1/1	**75.4 ± 8.2%**
Random forest	200 ms	2/1	75.0 ± 5.1%
KNN	200 ms	2/1	61.1 ± 3.4%
SVM	200 ms	2/1	67.2 ± 5.2%

The bold indicates better result.

The methods used for comparison include LSTM + CNN (He et al., 2018), k-nearest neighbor (KNN), support vector machine (SVM), random forest, and convolutional neural network (CNN) (Atzori et al., 2016). In all experiments, our method was second best in all methods and only 0.2% lower than the best. What is more, our method showed more stable results (2.3% fluctuations) than others.

**FIGURE 7:**
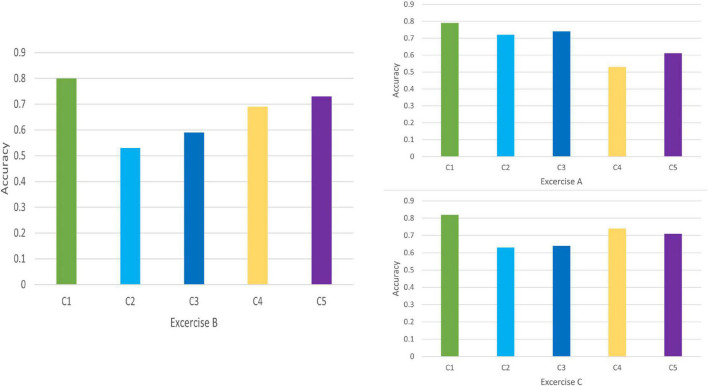
Feature discrimination results for DB1.

**FIGURE 8:**
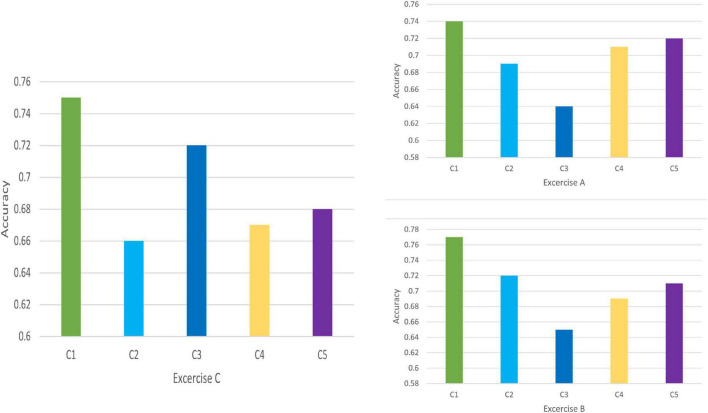
Feature discrimination results for DB2.

Discrimination results for Exercise A, Exercise B, and Exercise C in DB1 and DB2 is shown in Figures 7, 8, respectively. For each exercise, we compare feature combinations from layer 1–4. Detail feature combinations is described in Table 5. Tables 6–8 list the classification accuracy with different feature combinations for DB1, respectively.

**TABLE 5:** Feature combinations.

*C1*	(*z*^1^,*z*^2^,*z*^3^,*z*^4^)
*C2*	(*z*^2^,*z*^3^,*z*^4^)
*C3*	(*z*^1^,*z*^3^,*z*^4^)
*C4*	(*z*^1^,*z*^2^,*z*^4^)
*C5*	(*z*^1^,*z*^2^,*z*^3^)

**TABLE 6:** Classification results with different feature combinations for Exercise A.

Feature Combinations	Accuracy	Discrimination (C1-Accuracy)
C1	0.79	0
C2	0.72	0.07
C3	0.74	**0.05**
C4	0.53	**0.26**
C5	0.61	0.18

The bold values mean the lowest and highest discrimination values.

**TABLE 7:** Classification results with different feature combinations for Exercise B.

Feature Combinations	Accuracy	Discrimination (-C1)
C1	0.8	0
C2	0.53	**0.27**
C3	0.59	0.21
C4	0.69	0.11
C5	0.73	**0.07**

The bold values mean the lowest and highest discrimination values.

**TABLE 8:** Classification results with different feature combinations for Exercise C.

Feature Combinations	Accuracy	Discrimination (-C1)
C1	0.82	0
C2	0.63	**0.19**
C3	0.64	0.18
C4	0.74	**0.08**
C5	0.71	0.11

The bold values mean the lowest and highest discrimination values.

Discrimination value in Tables 6–8 measures the representation capability of feature in each layer. The higher the value, the better the feature representation ability. In Exercise A, C4 obtains the highest discrimination value, which means feature *z*^3^ plays the most import role in Exercise A. Similarly, feature *z*^2^ makes little difference in Exercise A.

## Conclusion

In this manuscript, we propose an Unsupervised Layer-wise Feature Extraction Algorithm (LFEA) to perform the sEMG signal processing and downstream classification tasks. The model contains three core modules: Information Compression Module (ICM), Information Expression Module (IEM) and Information Separation Module (ISM), that ensure that the learning representation is compact, informative and disentangled. We further use a layer-wise optimization procedure to reduce the computation cost and avoid some optimization problem, like vanishing and exploding gradient. Experimentally, we also verify that the untangling effect and downstream classification tasks give better results.

In the future, we hope to combine the advantages of supervised and unsupervised to build a semi-supervised learning framework that can be adapted to more scenarios.

## Data availability statement

The original contributions presented in this study are included in the article/supplementary material, further inquiries can be directed to the corresponding author.

## Author contributions

ML and ZL contributed to the conception and design of the study. FZ organized the database. JG performed the statistical analysis. ML and ST wrote the first draft of the manuscript. All authors contributed to the manuscript revision, read, and approved the submitted version.

## References

[B1] Abdi H. Williams L. J. (2010). Principal component analysis. *Wiley Interdiscip. Rev.* 2 433–459. 10.1002/wics.101

[B2] Alemi A. A. Fischer I. Dillon J. V. Murphy K. (2016). Deep variational information bottleneck. *arXiv.* [preprint]. arXiv:1612.00410.

[B3] Atzori M. Cognolato M. Müller H. (2016). Deep learning with convolutional neural networks applied to electromyography data: A resource for the classification of movements for prosthetic hands. *Front. Neurorobot.* 10:9. 10.3389/fnbot.2016.00009 27656140 PMC5013051

[B4] Atzori M. Gijsberts A. Castellini C. Caputo B. Hager A. G. M. Elsig S. (2014). Electromyography data for non-invasive naturally-controlled robotic hand prostheses. *Sci. Data* 1:140053. 10.1038/sdata.2014.53 25977804 PMC4421935

[B5] Belghazi M. I. Baratin A. Rajeshwar S. Ozair S. Bengio Y. Courville A. (2018). “Mutual information neural estimation,” in *International Conference on Machine Learning*, 531–540. (Stockholmsmässan, Stockholm Sweden)

[B6] Belkin M. Niyogi P. (2003). Laplacian eigenmaps for dimensionality reduction and data representation. *Neural Comput.* 15 1373–1396. 10.1162/089976603321780317

[B7] Bell A. J. Sejnowski T. J. (1995). An information-maximization approach to blind separation and blind deconvolution. *Neural Comput.* 7 1129–1159. 10.1162/neco.1995.7.6.1129 7584893

[B8] Bengio Y. Courville A. Vincent P. (2013). Representation learning: A review and new perspectives. *IEEE Trans. Pattern Anal. Mach. Intell.* 35 1798–1828. 10.1109/TPAMI.2013.50 23787338

[B9] Burgess C. P. Higgins I. Pal A. Matthey L. Lerchner A. (2018). Understanding disentangling ß in vae. *arXiv* [Preprint]. arXiv:1804.03599.

[B10] Chen R. T. Li X. Grosse R. B. Duvenaud D. K. (2018). Isolating sources of disentanglement in variational autoencoders. *Adv. Neural Inf. Proc. Syst.* [Preprint]. arXiv:1802.04942.

[B11] Cheng P. Min M. R. Shen D. Malon C. Zhang Y. Li Y. (2020b). Improving disentangled text representation learning with information-theoretic guidance. *arXiv* [preprint]. arXiv:2006.00693. 10.18653/v1/2020.acl-main.673

[B12] Cheng P. Hao W. Carin L. (2020a). Estimating Total Correlation with Mutual Information Bounds. *arXiv* [Preprint]. arXiv:2011.04794.

[B13] Gijsberts A. Atzori M. Castellini C. Müller H. Caputo B. (2014). Measuring movement classification performance with the movement error rate. *IEEE Trans. Neural Syst. Rehabil. Eng*. 89621, 735–744. 10.1109/TNSRE.2014.2303394 24760932

[B14] Gonzalez-Garcia A. Van De Weijer J. Bengio Y. (2018). Image-to-image translation for cross-domain disentanglement. *Adv. Neural Inf. Proc. Syst.* 31 1287–1298 34606453

[B15] Goodfellow I. Pouget-Abadie J. Mirza M. Xu B. Warde-Farley D. Ozair S. (2014). Generative adversarial nets. In *Advances in neural information processing systems* (Berlin: Springer), 2672-2680.

[B16] Hassani K. Khasahmadi A. H. (2020). Contrastive multi-view representation learing on graphs. *arXiv.* [Preprint]. arXiv:2006.05582.

[B17] He K. Zhang X. Ren S. Sun J. (2016). “Deep residual learning for image recognition,” in *Proceedings of the IEEE Conference on Computer Vision and Pattern Recognition*, (Washington, DC: IEEE Computer Society), 770–778. 10.1109/CVPR.2016.90

[B18] He Y. Fukuda O. Bu N. Okumura H. Yamaguchi N. (2018). “Surface emg pattern recognition using long short-term memory combined with multilayer perceptron,” in *2018 40th Annual International Conference of the IEEE Engineering in Medicine and Biology Society (EMBC)*, (Jeju Island: IEEE), 5636–5639. 10.1109/EMBC.2018.8513595 30441614

[B19] Higgins I. Matthey L. Pal A. Burgess C. Glorot X. Botvinick M. (2016). “Beta-VAE: Learning basic visual concepts with a constrained variational framework,” in *Proceedings of the international conference on learning representations*.

[B20] Hochreiter S. Schmidhuber J. (1997). Long short-term memory. *Neural Comput.* 9 1735–1780. 10.1162/neco.1997.9.8.1735 9377276

[B21] Howard A. G. Zhu M. Chen B. Kalenichenko D. Wang W. Weyand T. (2017). Mobilenets: Efficient convolutional neural networks for mobile vision applications. *arXiv.* [Preprint]. arXiv:1704.04861.

[B22] Hsu W. N. Zhang Y. Glass J. (2017). Unsupervised learning of disentangled and interpretable representations from sequential data. *Adv. Neural Inf. Proc. Syst.* [Preprint]. arXiv:1709.07902.

[B23] Izenman A. J. (2013). “Linear discriminant analysis,” in *Modern multivariate statistical techniques*, (New York, NY: Springer), 237–280. 10.1007/978-0-387-78189-1_8

[B24] Jeon I. Lee W. Pyeon M. Kim G. (2021). “Ib-gan: Disengangled representation learning with information bottleneck generative adversarial networks,” in *Proceedings of the AAAI Conference on Computer Vision and Pattern Recognition*, 7926–7934.

[B25] Kim H. Mnih A. (2018). “Disentangling by factorising,” in *International Conference on Machine Learning*, 2649–2658.

[B26] Kingma D. P. Welling M. (2013). Auto-encoding variational bayes. *arXiv.* [Preprint]. arXiv:1312.6114.1.

[B27] LeCun Y. Touresky D. Hinton G. Sejnowski T. (1988). “A theoretical framework for back-propagation,” in *In Proceedings of the 1988 Connectionist Models Summer School*, Vol. 1 21–28.

[B28] Liu Z. Li M. Han C. (2021). Blocked and Hierarchical Disentangled Representation From Information Theory Perspective. *arXiv.* [preprint]. arXiv:2101.08408.

[B29] Matsuda Y. Yamaguchi K. (2003). “The InfoMin criterion: An information theoretic unifying objective function for topographic mappings,” in *Artificial Neural Networks and Neural Information Processing—ICANN/ICONIP 2003*, (Berlin: Springer), 401–408. 10.1007/3-540-44989-2_48

[B30] Myung I. J. (2003). Tutorial on maximum likelihood estimation. *J. Math. Psychol.* 47 90–100. 10.1016/S0022-2496(02)00028-7

[B31] Richard M. D. Lippmann R. P. (1991). Neural network classifiers estimate Bayesian a posteriori probabilities. *Neural Comput.* 3 461–483. 10.1162/neco.1991.3.4.461 31167331

[B32] Shannon C. E. (2001). A mathematical theory of communication. *GetMobile* 5 3–55. 10.1145/584091.584093

[B33] Shwartz-Ziv R. Tishby N. (2017). Opening the black box of deep neural networks via information. *arXiv.* [Preprint]. arXiv:1703.00810.

[B34] Tenenbaum J. B. Silva V. D. Langford J. C. (2000). A global geometric framework for nonlinear dimensionality reduction. *Science* 290 2319–2323. 10.1126/science.290.5500.2319 11125149

[B35] Thomas M. T. C. A. J. Joy A. T. (2006). *Elements of information theory.* Hoboken, NJ: Wiley-Interscience.

[B36] Tishby N. Zaslavsky N. (2015). “Deep learning and the information bottleneck principle,” in *2015 IEEE Information Theory Workshop (ITW)*, (Jeju Island: IEEE), 1–5. 10.1109/ITW.2015.7133169

[B37] Tishby N. Pereira F. C. Bialek W. (2000). The information bottleneck method. *arXiv.* [Preprint]. physics/0004057.

[B38] Xing K. Ding Z. Jiang S. Ma X. Yang K. Yang C. (2018). “Hand gesture recognition based on deep learning method,” in *2018 IEEE Third International Conference on Data Science in Cyberspace (DSC)*, (Jeju Island: IEEE), 542–546. 10.1109/DSC.2018.00087

[B39] Yingzhen L. Mandt S. (2018). “Disentangled sequential autoencoder,” in *International Conference on Machine Learning*, 5670–5679.

[B40] Zbontar J. Jing L. Misra I. Lecun Y. Deny S. (2021). Barlow twins: Self-supervised learning via redundancy reduction. *Int. Conference Mach. Learn.* 139 12310–12320.

[B41] Zhai X. Jelfs B. Chan R. H. Tin C. (2017). Self-recalibrating surface EMG pattern recognition for neuroprosthesis control based on convolutional neural network. *Front. Neurosci.* 11:379. 10.3389/fnins.2017.00379 28744189 PMC5504564

